# Inter-rater Agreement of End-of-shift Evaluations Based on a Single Encounter

**DOI:** 10.5811/westjem.2016.12.32014

**Published:** 2017-03-07

**Authors:** Steven Warrington, Michael Beeson, Amber Bradford

**Affiliations:** * Kaweah Delta Medical Center, Department of Emergency Medicine, Visalia, California; † Akron General Medical Center, Department of Emergency Medicine, Akron, Ohio

## Abstract

**Introduction:**

End-of-shift evaluation (ESE) forms, also known as daily encounter cards, represent a subset of encounter-based assessment forms. Encounter cards have become prevalent for formative evaluation, with some suggesting a potential for summative evaluation. Our objective was to evaluate the inter-rater agreement of ESE forms using a single scripted encounter at a conference of emergency medicine (EM) educators.

**Methods:**

Following institutional review board exemption, we created a scripted video simulating an encounter between an intern and a patient with an ankle injury. That video was shown during a lecture at the Council of EM Residency Director’s Academic Assembly with attendees asked to evaluate the “resident” using one of eight possible ESE forms randomly distributed. Descriptive statistics were used to analyze the results with Fleiss’ kappa to evaluate inter-rater agreement.

**Results:**

Most of the 324 respondents were leadership in residency programs (66%), with a range of 29–47 responses per evaluation form. Few individuals (5%) felt they were experts in assessing residents based on EM milestones. Fleiss’ kappa ranged from 0.157 – 0.308 and did not perform much better in two post-hoc subgroup analyses.

**Conclusion:**

The kappa ranges found show only slight to fair inter-rater agreement and raise concerns about the use of ESE forms in assessment of EM residents. Despite limitations present in this study, these results and a lack of other studies on inter-rater agreement of encounter cards should prompt further studies of such methods of assessment. Additionally, EM educators should focus research on methods to improve inter-rater agreement of ESE forms or other evaluating other methods of assessment of EM residents.

## INTRODUCTION

End-of-shift evaluation (ESE) forms, also known as daily encounter cards, are useful for assessing performance in a non-simulated clinical environment. While many other methods exist, such as the mini-clinical evaluation exercise and the Standardized Direct Observation Assessment Tool, the use of ESE forms has become more common.^1^–^4^ ESE forms are used in emergency medicine (EM), internal medicine, surgery, obstetrics and gynecology, and pediatrics.^5^–^8^ In addition to assessing medical students and residents, they have also been used for evaluation of faculty.^9^

Generation of feedback, feasibility to implement, minimal recall, and acceptance as a method of evaluation are reasons that ESE forms have become so popular. Some authors found increased feedback after the implementation of encounter cards with students that is inclusive of multiple domains.^2^,^5^,^7^,^8^ Others have found encounter cards practical to implement for individual encounters and daily encounters.^10^ Individuals do not feel the time required is burdensome, with multiple authors noting a high completion rate. Both students and faculty are comfortable using ESE forms.^6^,^9^

Some issues have been raised on using encounter cards for evaluation. One of them is conflicting evidence on learner satisfaction with the feedback generated.^10^ Another is that assessments using ESE cards suffer from leniency bias, which may lead to inaccurate evaluation.^2^ Finally, data entry after completing an evaluation card may add administrative time not initially planned.^7^

As the ESE form represents an evaluation and may have a role in summative assessment, the measurement characteristics such as inter-rater reliability and internal consistency should be considered.^11^ Aspects of an evaluation form’s internal structure include inter-rater reliability and inter-rater agreement.^12^ ESE forms have been shown to have acceptable inter-rater reliability assessing students.^13^ While inter-rater reliability and inter-rater agreement may coexist, an acceptable inter-rater reliability doesn’t guarantee acceptable inter-rater agreement, making it necessary to evaluate the inter-rater agreement as well.^14^

The primary objective of this study was to evaluate the inter-rater agreement of ESE forms using a single encounter. We hypothesized that there would be a high rate of inter-rater agreement.

## METHODS

### Development of ESE forms

**Figure 1:**
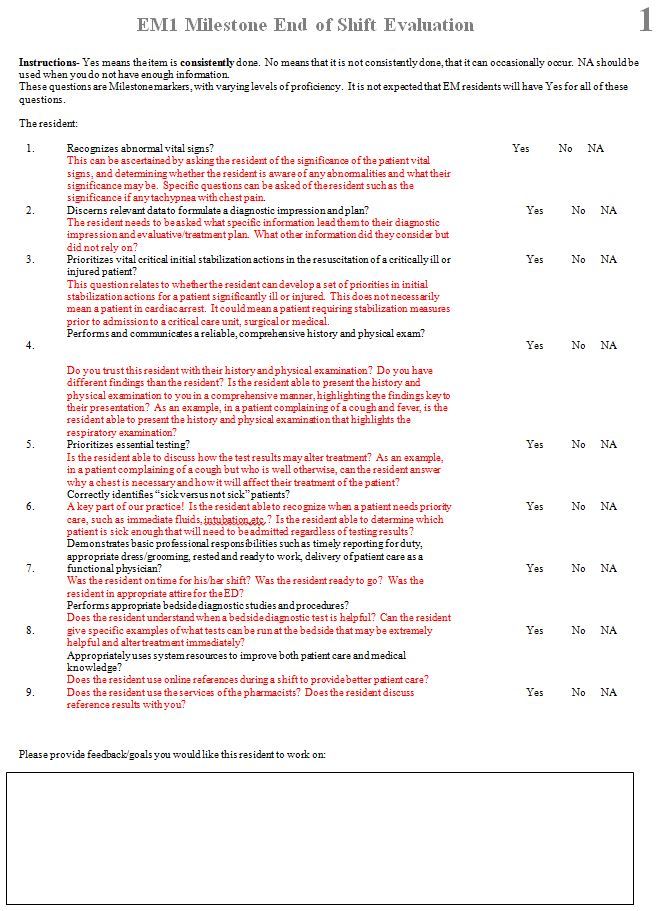
One end-of-shift evaluation form for emergency medicine interns.

**Figure 2:**
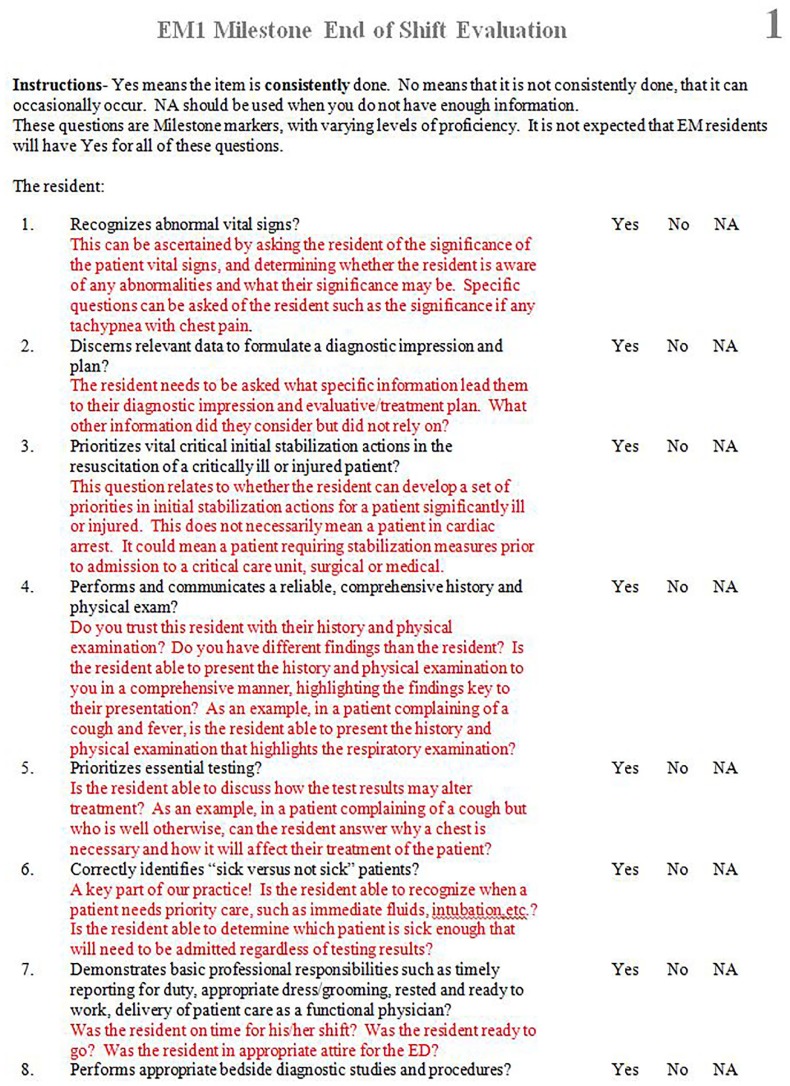

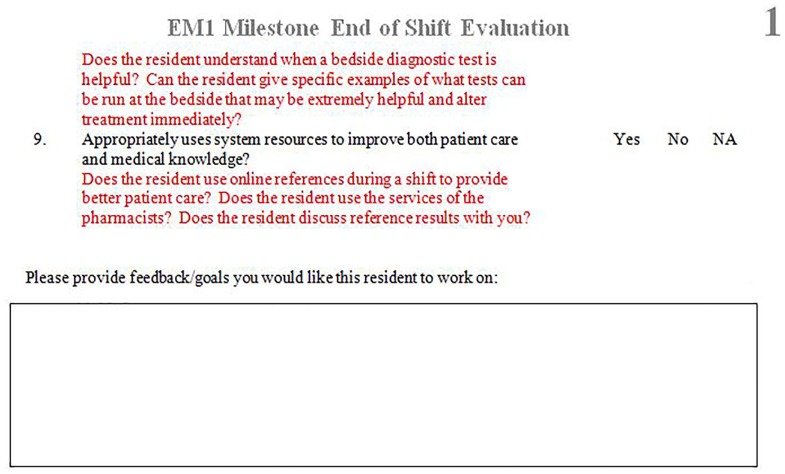
One scoring anchor for an end-of-shift evaluation form.

We developed a set of eight ESE forms for interns and eight for more senior residents to address the new assessment needs of the EM milestones.^15^,^16^ Multiple forms were used instead of one due to the number of questions necessary to assess each milestone and subcompetency. Each question used language directly from individual milestones since the EM Milestones Project involved multiple forms of validity evidence.^17^,^18^ We developed a separate set of forms for interns and senior residents due to the different milestone levels. A section to provide open-ended feedback was also included. Answer choices for each question on the form were “yes,” “no,” or “not applicable,” and were further explained with scoring anchors. Examples of a form and scoring anchor are in Figures 1 and 2 respectively. These forms were then shared and implemented at multiple residency programs across the country. Anecdotal evidence from the implementation showed them to be both feasible to implement and easy to use. The forms used in this study to assess interns, collectively capture 76 data points from 16 of the 23 subcompetencies. The six procedural subcompetencies were purposefully left out due to the ability to assess those subcompetencies through existing formats. The medical knowledge subcompetency was also left out as its milestones could not be evaluated from ESE forms (e.g., “Passes national licensing examinations”).^16^

**Population Health Research Capsule**

What do we already know about this issue?

End-of-shift evaluation forms are a common method of evaluating learners and faculty. Some evidence of validity has been demonstrated with prior research.

What was the research question?

What is the inter-rater agreement of one set of end-of-shift evaluation forms using a single encounter?

What was the major finding of the study?

Inter-rater agreement was only slight to fair when using one set of end-of-shift evaluation forms.

How does this improve population health?

This study identifies lack of one aspect of validity evidence for a common assessment tool used to evaluate EM residents’ competency.

### Standardized video

We developed a video using a scripted encounter simulating an EM intern evaluating a patient with an ankle injury. The script for the encounter was based on the ESE forms for assessing interns to ensure approximately equal representation of answers for “yes,” “no,” and “not applicable.”

### Data collection

Following institutional review board approval at the authors’ institution, the standardized video was played during a lecture on EM milestone assessment at the 2013 Council of Emergency Medicine Residency Directors Academic Assembly. Individuals in the lecture were randomly given one of the eight forms available for assessing an intern based on where they sat at the beginning of the lecture. The attendees were asked to complete their ESE form based on the encounter in the video. Forms given included scoring anchors attached and were identical to the forms developed, with the exception of added demographic data on the respondent’s role in their residency program and their perception of their own knowledge level on the EM milestones. Completion was voluntary and anonymous as there was no personal or program identifying information.

### Analysis of data

We evaluated the data obtained by descriptive statistics with inter-rater agreement tested on each form using Fleiss’ kappa using listwise deletion for incomplete datasets. Two post-hoc subgroups were analyzed for inter-rater agreement as follows:

After an initially low kappa, we excluded from analysis data from program coordinators and those with self-identified minimal knowledge. Inter-rater agreement was re-calculated using Fleiss’ kappa as post-hoc analysis 1. This was done after finding only fair inter-rater agreement to determine if those not familiar with the milestones or assessing residents affected the data.

We used post-hoc analysis 2 to determine the inter-rater agreement of each competency’s milestones from all forms combined; this was done to determine if inter-rater agreement using an ESE form was partially dependent on domain evaluated. In calculating kappa for each domain the data required adjustment due to each set of forms having a different number of respondents (range 29–47). As Fleiss’ kappa does not require each rater to rate each item, we grouped all items related to a competency from each of the eight forms. Then items with less than 47 raters were assigned a null value to allow for Fleiss’ kappa to be completed, as it requires the same total number of raters. To address the potential bias created by including the average of the null category, which was inevitably low, we then recalculated the average kappa without the null kappa. Of note, the competency “Practice-Based Learning and Improvement” did not have a kappa calculated, as there was only one milestone for evaluation associated with it on the eight forms.

We performed data analysis using the Real Statistics Resource Pack software ([Release 4.3] Copyright 2013 – 2015, Charles Zaiontz [www.real-statistics.com]).^19^

## RESULTS

### Descriptive results

A total of 324 forms were turned in with 318 (98.1%) providing information on roles within the program, 313 (96.6%) providing self-ranking of knowledge on the EM milestones, and 309 (95.4%) having all ESE questions answered. Most respondents self-identified as assistant/associate program director (38%), followed by program directors (28%), and other non-program coordinator individuals (24%), and finally program coordinators (11%). Over half of the respondents (58%) identified themselves as “knowledgeable but not expert,” with approximately one third (37%) characterizing their knowledge as “minimal,” while few (5%) labeled themselves as “expert.”

### Inter-rater agreement

Each of the eight forms’ kappa was determined based on data collected after listwise deletion to address incomplete forms and ranged from 0.157 – 0.308, with number of respondents per form listed in Table. Removal of data from program coordinators and those who self-identified as having minimal knowledge on the EM milestones did not significantly change the results with a kappa range = 0.158 – 0.358 (see Table). Finally, average kappa by domain (Patient Care, Interpersonal and Communication Skills, Professionalism, and Systems-Based Practice) instead of form were calculated and ranged from 0.155 – 0.222 (Table).

## DISCUSSION

Using generally accepted interpretations of kappa the results show there was slight to fair agreement among observers of a single scripted resident-patient interaction.^20^ Taking out individuals twho self-identified to not have much knowledge on the EM milestones and program coordinators who were not expected to have much knowledge with assessment of residents did not result in a significant increase in inter-rater agreement. Further analysis of the data showed similarly disappointing inter-rater agreement using an ESE form for individual domains.

The most concerning ramification of this study is the need consider the low inter-rater agreement as one threat to validity evidence of ESE forms and encounter cards. While inter-rater agreement may not be important if the form is being used to collect feedback, it is important to consider if the form is being used as a formal evaluation of learners. Consideration of this threat, as with all other validity evidence, should be used when educators are selecting assessment tools useful for the situation and setting. One example is when multiple individuals will be assessed infrequently by a large number of raters. In that instance the evidence for acceptable inter-rater reliability using encounter cards may be overcome by the threat of poor inter-rater agreement.^13^ Additionally, programs using ESE forms as part of a summative assessment, as suggested by others, should consider further evaluation of their own ESE form’s validity evidence.^11^

A second ramification of this study is the need for further research on methods to improve inter-rater agreement of ESE forms. As these forms have become popular the ability to improve testing characteristics using them would make them more useful. Methods to be studied could include pre-training faculty on forms, focused faculty development on assessment, and evaluation of scoring-anchor characteristics.

## LIMITATIONS

We noted multiple limitations regarding our study. First, while it was conducted with individuals who were expected to have experience in assessment of residents, the lack of training on the specific ESE forms used was a limitation and may have biased the results obtained. Importantly, this was recognized by the authors, but as some institutions implement such evaluation methods without pre-training faculty the study was felt to be representative of the authors’ institutions (i.e., without pre-training faculty). While some residency programs provide significant training to all faculty prior to implementation, not all residency programs have that capability, and so this study represents the potential inter-rater agreement at such institutions. Evaluation of inter-rater agreement of ESE forms completed by individuals who have undergone training prior to their use may yield different results and represents potential secondary research.

Another limitation of the study was the use of a single recorded encounter despite the ESE forms being intended for assessment following the completion of a shift in the emergency department. Due to the setting being a session at a national conference, and the inherent time limitations associated with that, the authors did not feel more than one recorded encounter would be able to be shown and evaluated. While it can be hypothesized that our ESE form could translate to use for one encounter, it is still a limitation. Studying the ESE form’s inter-rater agreement based on a full shift, or multiple patient encounters, was not feasible in the setting chosen.

A third limitation of the study and using these forms for evaluation purposes is the fact that eight separate questionnaires for seniors, and another eight separate questionnaires for interns, were used due to the number of questions that would be required if only one form were used. Each individual form only targets certain domains and sub-competencies and in doing so limits when data points are collected on learners and makes the evaluation of such forms more difficult. Regardless, it was felt necessary due to the potential for fatigue bias and potential that faculty may be more likely to complete evaluations in this format compared to a single form with over 50 questions per evaluation.

A final limitation of this study was the possibility that the domains planned for assessment in the EM milestones may not have translated into the questions on the ESE forms developed. While language was used directly from the EM milestones, validity evidence from their development doesn’t necessarily translate to validity evidence of the forms. No strict guidelines were used, aside from following the EM milestones, in the development of the ESE forms.

## CONCLUSION

This study adds to the current literature on assessment in emergency medicine using ESE forms by documenting evidence of their slight to fair inter-rater agreement. Its importance stems from educators’ needs to identify assessment instruments that will perform at an acceptable level in their setting for a chosen purpose. Educators must consider the low inter-rater agreement of ESE forms when choosing them as an assessment tool.

## Figures and Tables

**Table:** Kappa for each analysis of end-of-shift evaluation forms.

Analysis	Number of forms	Kappa
Original data	324	0.223
ESE form 1	44	0.202
ESE form 2	29	0.308
ESE form 3	43	0.248
ESE form 4	39	0.199
ESE form 5	47	0.157
ESE form 6	38	0.301
ESE form 7	44	0.213
ESE form 8	40	0.159
Post-hoc subset 1	186	0.232
ESE form 1	22	0.175
ESE form 2	16	0.304
ESE form 3	26	0.277
ESE form 4	25	0.228
ESE form 5	26	0.158
ESE form 6	24	0.358
ESE form 7	26	0.177
ESE form 8	21	0.18
Post-hoc subset 2	N/A	0.184
Patient care	N/A	0.202
Interpersonal and communication skills	N/A	0.222
Professionalism	N/A	0.155
Systems-based practice	N/A	0.156

Original Data: Fleiss’ kappa for each form without any exclusions

Post-hoc Subset 1: Program coordinators and those self-identified with minimal knowledge excluded from analysis.

Post-hoc Subset 2: Fleiss’ kappa calculated by domain and not by form.
